# Supplementary material for: Low-temperature synthesis and investigation into the formation mechanism of high quality Ni-Fe layered double hydroxides hexagonal platelets
Source: Sci Rep. 2018 Mar 8;8:4179. doi: 10.1038/s41598-018-22630-0 (PMC5843585; doi:10.1038/s41598-018-22630-0)
Supplement: Supplementary file 1 — Supplementary information [file 41598_2018_22630_MOESM1_ESM.doc]

Supplementary information

Low-temperature synthesis and investigation into the formation mechanism of high quality Ni-Fe layered double hydroxides hexagonal platelets

Sonia Jaśkaniec,^a,b^ Christopher Hobbs,^b,c^ Andrés Seral-Ascaso,^b,c^ João Coelho,^a,b^ Michelle P. Browne,^d^ Daire Tyndall,^a,b^ Takayoshi Sasaki^e^ and Valeria Nicolosi^a,b,c^,*

^a^School of Chemistry, Trinity College Dublin, Ireland

^b^CRANN&AMBER, Trinity College Dublin, Ireland

^c^School of Physics, Trinity College Dublin, Ireland

^d^School of Chemistry, Queens University Belfast, Ireland

^e^National Institute for Materials Science, Tsukuba, Japan

*nicolov@tcd.ie


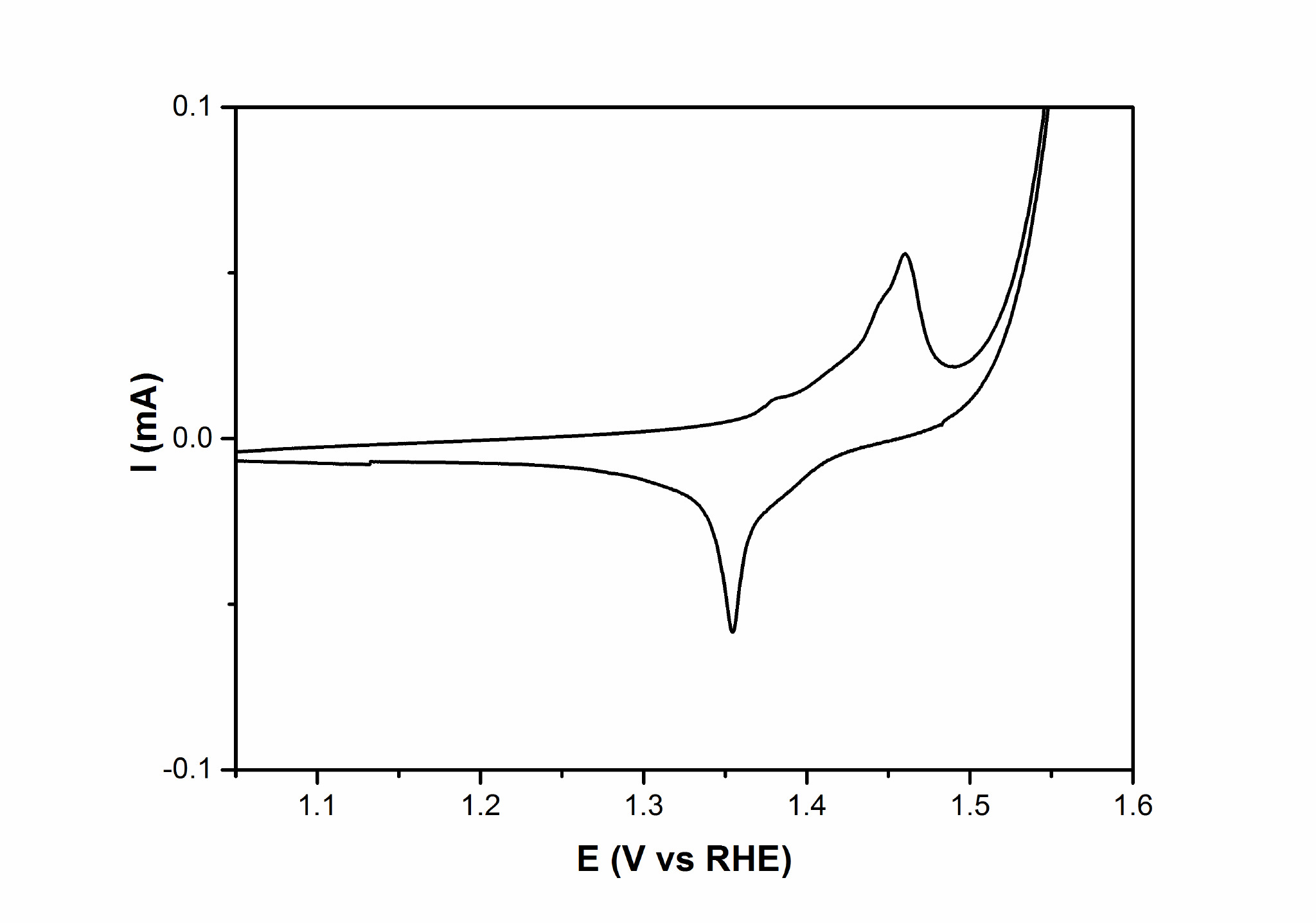


**Fig. S1** Cyclic voltammetry of Ni foam in 1 M KOH

**Fig. S2** UV-Vis spectra of Ni(NO_3_)_2_ and Fe(NO_3_)_3_ dissolved in water.

Fig. S3 Absorbance at 350 nm at different stirring time at room temperature.


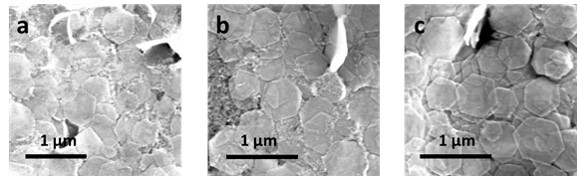


**Fig. S4** SEM micrographs of Ni_x_Fe_y_ LDH formed after a) 4 hours, b) 12 hours and c) 24 hours of heating*.*
